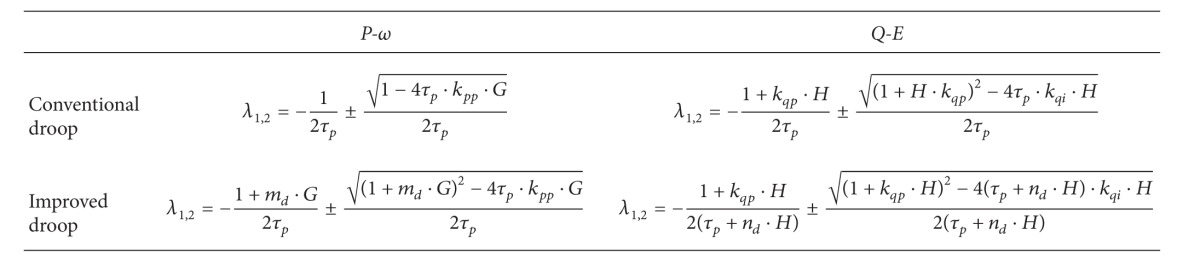# Analysis and Experimental Verification of New Power Flow Control for Grid-Connected Inverter with LCL Filter in Microgrid

**DOI:** 10.1155/2014/263590

**Published:** 2014-02-04

**Authors:** Herong Gu, Yajuan Guan, Huaibao Wang, Baoze Wei, Xiaoqiang Guo

**Affiliations:** Key Lab of Power Electronics for Energy Conservation and Motor Drive of Hebei Province, Yanshan University, Qinhuangdao 066004, China

## Abstract

Microgrid is an effective way to integrate the distributed energy resources into the utility networks. One of the most important issues is the power flow control of grid-connected voltage-source inverter in microgrid. In this paper, the small-signal model of the power flow control for the grid-connected inverter is established, from which it can be observed that the conventional power flow control may suffer from the poor damping and slow transient response. While the new power flow control can mitigate these problems without affecting the steady-state power flow regulation. Results of continuous-domain simulations in MATLAB and digital control experiments based on a 32-bit fixed-point TMS320F2812 DSP are in good agreement, which verify the small signal model analysis and effectiveness of the proposed method.

## 1. Introduction

The environmental concerns and electric utility deregulation promote the development of distributed generation (DG) in a rapid pace [1]. The high penetration of DG brings about a concept of the microgrid [2]. It is defined as a cluster of DG units (such as wind turbines and photovoltaics), storage devices, and loads, which can operate in the autonomous mode or grid-connected mode [2], and this paper focuses on the latter. Generally speaking, a grid-connected voltage-source inverter is used for the power flow control of DG unit in microgrid. During the last decades, the grid-connected inverter with a single inductor filter has been the prevalent choice [3]. However, a relatively large inductance has to be chosen to meet the existing harmonics emission standards such as IEEE 519 and IEEE 1547, mainly because of the high current ripple due to the switching mode inverter. The large inductance may bring on the large volume, high cost, and dynamic restriction of the system. An attractive solution is to replace the conventional filter with the inductance-capacitance-inductance (LCL) filter [4]. In this way, the current ripple attenuation (−60 dB) is more effective than the conventional one (−20 dB) even for a smaller inductance size, because the capacitor impedance is inversely proportional to the current frequency.

Power flow regulation of the grid-connected inverter with LCL filter can be mainly classified into three categories, namely, current control (CC), voltage control (VC), and power control (PC). Current control (CC) regulates the inverter current to track its reference, which is determined by the output power and grid voltage. A challenge of CC is how to achieve the fast and accurate current regulation with the passive or active damping, which is used to avoid the system instability resulted from the high-order LCL filter. Some lossless active-damping solutions have been presented such as the virtual resistor or multiloop feedback methods [5, 6]. However, it should be noted that the passive damping has to be used to ensure the system stability at the cost of power losses when the resonance frequency of LCL filter is outside the bandwidth of the closed loop system [7]. On the other hand, voltage control (VC) regulates the capacitor voltage to track its reference, which is determined by the output power and line impedance [8]. Although VC is more sensitive to system parameters and has a slower dynamic response than CC, it still remains to be investigated by many researchers. The reason for that is that VC can be easily utilized to provide the reliable support for the local sensitive loads when the utility is disconnected or interrupted [9]. But CC needs to be designed for the mode transfer from CC to VC to ensure the safety operation of local loads, during which the transient inrush may arise if the transfer mode control is not properly designed. Some improved VC methods have been reported in [10] aiming at operating in both grid-connected and grid-disconnected modes, but they focus on the single DG unit. In practical applications, especially in microgrid, multiple DG units may operate simultaneously, and these VC methods should be modified to share the power between DG units. In order to achieve the power sharing, the droop-based power control (PC) may be used [11], which has been well established for multi-inverter operation in autonomous mode. For grid-connection mode, a small modification of the conventional droop-based PC is needed [2]. In practice, however, this solution may suffer from the poor damping and slow transient response.

The contribution of this paper is to present a comprehensive small-signal model for the droop-based power control of the grid-connected inverter, from which the poor damping and slow transient response can be easily understood. And then, a new solution is presented for improving the poor damping and transient performances without affecting the steady-state regulation of the power flow. Finally, the experimentally comparative evaluations are carried out to highlight the contribution.

## 2. Model and Analysis of Power Flow Control

**Figure 1:**
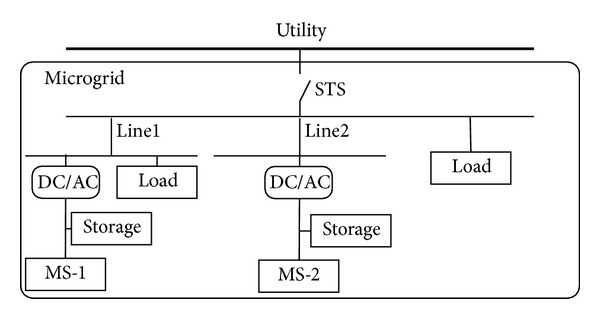
Microgrid configuration.

Figure 1 illustrates the schematic diagram of the microgrid. It comprises of the primary microsources (MS) with optional energy storages and dc/ac inverters. The inverters can provide an interface for the flexible functions such as power flow control and power quality improvement. The inverter output may either feed the local loads independently in autonomous mode or in conjunction with the electric utility by static switch (STS) in grid connected mode. This paper will focus on the latter mode.

**Figure 2:**
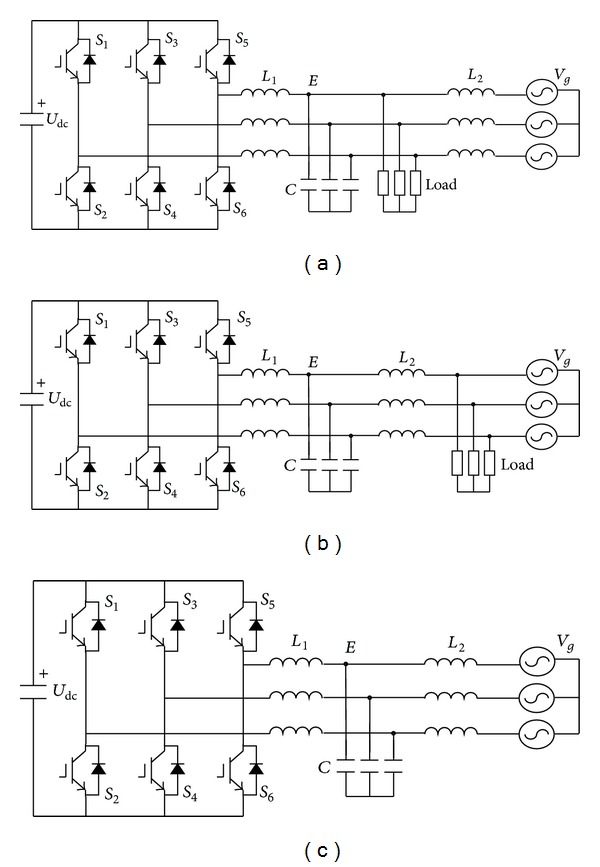
Grid-connected inverter with LCL filter configuration: (a) case I; (b) case II; (c) case III.

For simplicity, only one inverter is considered, and the analysis can be extended to multi-inverter cases. As shown in Figure 2, The configuration of the grid-connected inverter with LCL filter in microgrid has three typical patterns according to load locations. The first case [2] in Figure 2(a) has the better resonance damping than the latter two cases, because the load paralleled with the capacitor can be considered as the passive damping, which enhances the system stability. On the other hand, the load in Figure 2(b) will provide almost no passive damping because the terminal is clamped by the grid. And the second case [12] in Figure 2(b) can be simplified into the third case in Figure 2(c) from the viewpoint of the inverter output power flow control. In this paper, only the worst damping case in Figure 2(c) is investigated for highlighting the poor damping and slow transient response of the power flow control.

From Figure 2(c), it can be observed that the power flow control depends on the capacitor voltage, the grid voltage, and the inductance between them. The active and reactive powers can be expressed as follows [13]:

(1) $$\begin{array}{c} P=\frac{EV_{g}\sin\left(\delta -\delta_{g}\right)}{X}, \end{array}$$

(2) $$\begin{array}{c} Q=\frac{E^{2}-EV_{g}\cos\left(\delta -\delta_{g}\right)}{X}, \end{array}$$

where *E* and *δ* are the magnitude and angle of the capacitor voltage, respectively. *V*_*g*_ and *δ*_*g*_ *δ* are the magnitude and angle of the grid voltage, respectively. *X* is the impedance of *L*_2_.

In order to clarify the basic operation of the power flow control, the following equations are provided:

(3) $$\begin{array}{c} \frac{\partial P}{\partial \delta }=\frac{EV_{g}\cos\left(\delta -\delta_{g}\right)}{X},\\ \frac{\partial P}{\partial E}=\frac{V_{g}\sin\left(\delta -\delta_{g}\right)}{X},\\ \frac{\partial Q}{\partial \delta }=\frac{EV_{g}\sin\left(\delta -\delta_{g}\right)}{X},\\ \frac{\partial Q}{\partial E}=\frac{2E-V_{g}\cos\left(\delta -\delta_{g}\right)}{X}. \end{array}$$

In practice, the angle difference *δ* − *δ*_*g*_ is relatively small; that is, sin(*δ* − *δ*_*g*_) ≈ 0 and cos⁡(*δ* − *δ*_*g*_) ≈ 1. So, (3) can be rewritten as

(4) $$\begin{array}{c} \frac{\partial P}{\partial \delta }\approx \frac{EV_{g}}{X}, \end{array}$$

(5) $$\begin{array}{c} \frac{\partial P}{\partial E}\approx 0, \end{array}$$

(6) $$\begin{array}{c} \frac{\partial Q}{\partial \delta }\approx 0, \end{array}$$

(7) $$\begin{array}{c} \frac{\partial Q}{\partial E}\approx \frac{2E-V_{g}}{X}. \end{array}$$

From (4) to (7), it can be concluded that the active power flow regulation is more dependent on the capacitor voltage angle variation, while the reactive power flow is more sensitive to the capacitor voltage magnitude. Therefore, the conventional droop control can be obtained as follows:

(8) $$\begin{array}{c} \omega =\omega^{*}+k_{pp}\cdot \left(P^{*}-P\right), \end{array}$$

(9) $$\begin{array}{c} \delta -\delta_{g}=\int \left(\omega -\omega_{g}\right)\cdot dt, \end{array}$$

(10) $$\begin{array}{c} E=E^{*}+\left(k_{qp}+\frac{k_{qi}}{s}\right)\cdot \left(Q^{*}-Q\right). \end{array}$$

Note that the integral term is added in (10) to ensure the accurate reactive power flow control [2].

### 2.1. Model of Conventional Droop Control

The small-signal dynamics of the conventional *P*-*ω* droop can be obtained by linearizing (1), (8), and (9) at operation points *P*_*o*_, *δ*_*o*_, and *δ*_*go*_ as follows:

(11) Δω(s)=Δω∗(s)+kpp(P∗(s)−ΔP(s)),

(12) $$\begin{array}{c} \Delta P\left(s\right)=G\cdot \Delta \left(\delta \left(s\right)-\delta_{g}\left(s\right)\right), \end{array}$$

where *G* = (*E*_*o*_ · *V*_*go*_ · cos⁡⁡(*δ*_*o*_ − *δ*_*go*_))/*X*, *d*(Δ*δ*(*s*) − Δ*δ*_*g*_(*s*))/*dt* = Δ*ω*(*s*) − Δ*ω*_*g*_(*s*).

**Figure 3:**
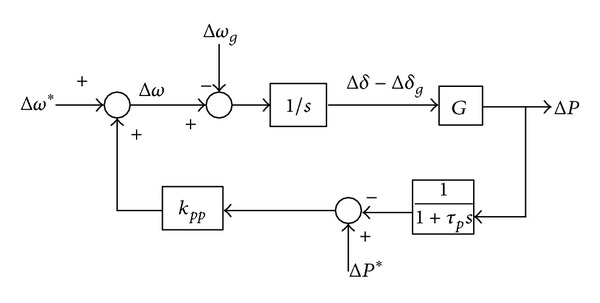
Small signal model of the conventional *P*-*ω* droop control.

The small signal model can be obtained based on (11) and (12). Consider the effect of the low pass filter on the power flow control; the small signal model of the conventional *P*-*ω* droop control can be obtained as shown in Figure 3.

From Figure 3, the active power Δ*P* can be expressed as

(13) ΔP(s)=G·(1+τps)τps2+s+kpp·G·Δω∗(s) −G·(1+τps)τps2+s+kpp·G·Δωg(s) +G·kpp·(1+τps)τps2+s+kpp·G·ΔP∗(s).

From (13), the characteristic equation and its eigenvalue can be obtained as

(14) $$\begin{array}{c} \tau_{p}s^{2}+s+k_{pp}\cdot G=0, \end{array}$$

(15) $$\begin{array}{c} \lambda_{1,2}=-\frac{1}{2\tau_{p}}\pm \frac{1}{2\tau_{p}}\cdot \sqrt{1-4\tau_{p}\cdot k_{pp}\cdot G}. \end{array}$$

In the same way, the small-signal dynamics of the conventional *Q*-*E* droop can be obtained by linearizing equations (2) and (10) at operation points *Q*_*o*_, *δ*_*o*_, and *δ*_*go*_ as follows:

(16) $$\begin{array}{c} \Delta E\left(s\right)=\Delta E^{*}\left(s\right)+\left(k_{qp}+\frac{k_{qi}}{s}\right)\cdot \left(\Delta Q^{*}\left(s\right)-\Delta Q\left(s\right)\right), \end{array}$$

(17) $$\begin{array}{c} \Delta Q\left(s\right)=F\cdot \Delta V_{g}\left(s\right)+H\cdot \Delta E\left(s\right), \end{array}$$

where *F* = −(*E*_*o*_ · cos⁡⁡(*δ*_*o*_ − *δ*_*go*_))/*X*, *H* = (2*E*_*o*_ − *V*_*go*_cos⁡⁡(*δ*_*o*_ − *δ*_*go*_))/*X*.

The small signal model can be obtained based on (16) and (17). Consider the effect of the low pass filter on the power flow control; the small signal model of the conventional *Q*-*E* droop control can be obtained as follows.

**Figure 4:**
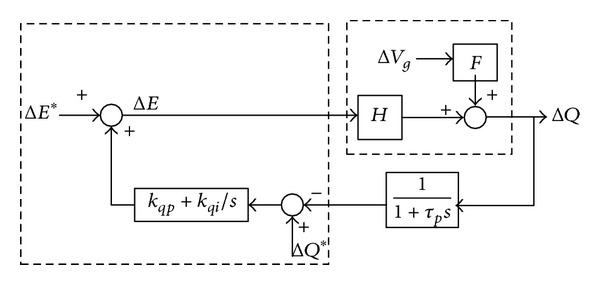
Small signal model of the conventional *Q*-*E* droop control.

From Figure 4, the reactive power Δ*Q* can be expressed as

(18) ΔQ(s)=H·s·(1+τps)τps2+(1+H·kqp)·s+kqi·H·ΔE∗(s) +F·s·(1+τps)τps2+(1+H·kqp)·s+kqi·H·ΔEg(s) +H·(kqp·s+kqi)·(1+τps)τps2+(1+H·kqp)·s+kqi·H·ΔQ∗(s).

From (18), the characteristic equation and its eigenvalue can be obtained as

(19) $$\begin{array}{c} \tau_{p}s^{2}+\left(1+H\cdot k_{qp}\right)\cdot s+H\cdot k_{qi}=0, \end{array}$$

(20) $$\begin{array}{c} \lambda_{1,2}=-\frac{1+H\cdot k_{qp}}{2\tau_{p}}\pm \frac{1}{2\tau_{p}}\cdot \sqrt{{\left(1+H\cdot k_{qp}\right)}^{2}-4\tau_{p}\cdot k_{qi}\cdot H}. \end{array}$$

From the small signal model and the system eigenvalues of (15) and (20), it can be concluded that the dynamic responses of the power flow control mainly depend on the droop parameters (*k*_*pp*_, *k*_*qp*_) and the filter parameter (*τ*_*p*_). Note that the filter parameter *τ*_*p*_ should be carefully chosen in order not to interact with the inner control. On the other hand, the droop parameters *k*_*pp*_ and *k*_*qp*_ have to be designed with small values, because too large *k*_*pp*_ and *k*_*qp*_ will result in an unacceptable power variation when the grid experiences disturbances. Therefore, the system dynamic response cannot be optimized, and the problems of poor damping and slow transient response may arise.

In order to solve the problems of the conventional droop control, the improved solution is presented as follows:

(21) $$\begin{array}{c} \omega =\omega^{*}+k_{pp}\cdot \left(P^{*}-P\right)+m_{d}\cdot \frac{dP}{dt}, \end{array}$$

(22) $$\begin{array}{c} E=E^{*}+\left(k_{qp}+\frac{k_{qi}}{s}\right)\cdot \left(Q^{*}-Q\right)+n_{d}\cdot \frac{dQ}{dt}. \end{array}$$

### 2.2. Model of Improved Droop Control

From the model in the previous section and (21), the small signal model of the improved *P*-*ω* droop control can be obtained as follows.

**Figure 5:**
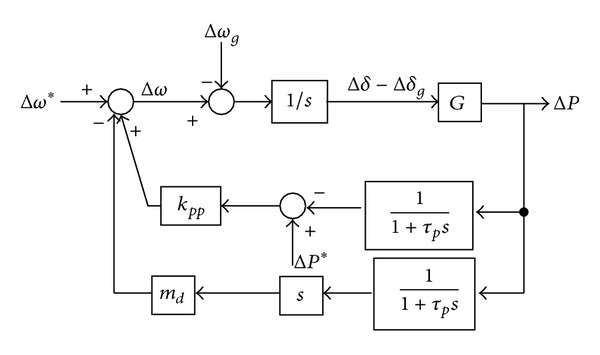
Small signal model of the improved *P*-*ω* droop control.

From Figure 5, the active power Δ*P* can be expressed as

(23) ΔP(s)=G·(1+τps)τps2+(1+md·G)·s+kpp·G·Δω∗(s) −G·(1+τps)τps2+(1+md·G)·s+kpp·G·Δωg(s) +kpp·G·(1+τps)τps2+(1+md·G)·s+kpp·G·ΔP∗(s).

From (23), the characteristic equation and its eigenvalue can be obtained as

(24) $$\begin{array}{c} s^{2}\tau_{p}+\left(1+m_{d}\cdot G\right)s+k_{pp}\cdot G=0, \end{array}$$

(25) $$\begin{array}{c} \lambda_{1,2}=-\frac{1+m_{d}\cdot G}{2\tau_{p}}\pm \frac{1}{2\tau_{p}}\cdot \sqrt{{\left(1+m_{d}\cdot G\right)}^{2}-4\tau_{p}\cdot k_{pp}\cdot G}. \end{array}$$

Similarly, the small signal model of the improved *Q*-*E* droop control can be obtained as follows.

**Figure 6:**
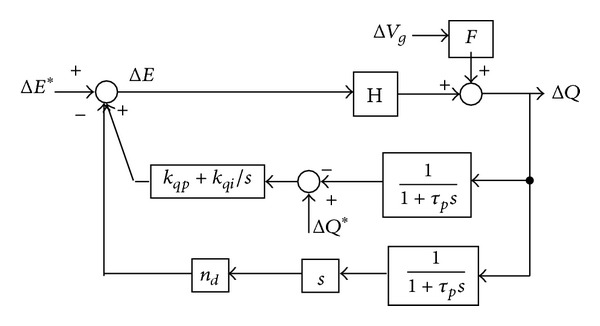
Small signal model of the improved *Q*-*E* droop control.

From Figure 6, the reactive power Δ*Q* can be expressed as

(26) ΔQ(s)=H·s·(1+τps)(nd·H+τp)·s2+(1+kqp·H)·s+kqi·H ·ΔE∗(s) +F·s·(1+τps)(nd·H+τp)·s2+(1+kqp·H)·s+kqi·H ·ΔEg(s) +H·(kqps+kqi)·(1+τps)(nd·H+τp)·s2+(1+kqp·H)·s+kqi·H ·ΔQ∗(s).

From (26), the characteristic equation and its eigenvalue can be obtained as

(27) $$\begin{array}{c} s^{2}\left(\tau_{p}+n_{d}\cdot H\right)+\left(1+k_{qp}\cdot H\right)s+k_{qi}\cdot H=0, \end{array}$$

(28) λ1,2=−1+kqp·H2(τp+nd·H)±12(τp+nd·H) ·(1+kqp·H)2−4(τp+nd·H)·kqi·H.

It is well known that the eigenvalue can reveal the system stability, damping, and transient response. Generally, the system stability can be confirmed if all the eigenvalues have negative real parts, and the oscillatory frequency in transient response depends on the imaginary parts of dominant complex-conjugate eigenvalues. Besides, the further the left half-plane eigenvalues are away from the imaginary axis, the faster the transient response is [3].  Table 1 presents the brief comparison of eigenvalues from different controls.

Compared with the eigenvalues of conventional droop control in Table 1, it can be observed that the proposed droop control has two additional parameters of *m*_*d*_ and *n*_*d*_, which can be used to improve the poor damping and slow transient response. The following will present the experimental verification.

## 3. Simulation and Experimental Results

**Figure 7:**
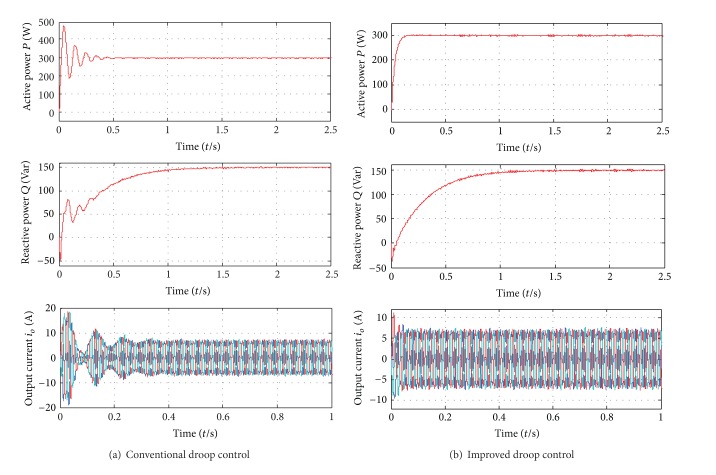
Simulation results of transient responses.

**Figure 8:**
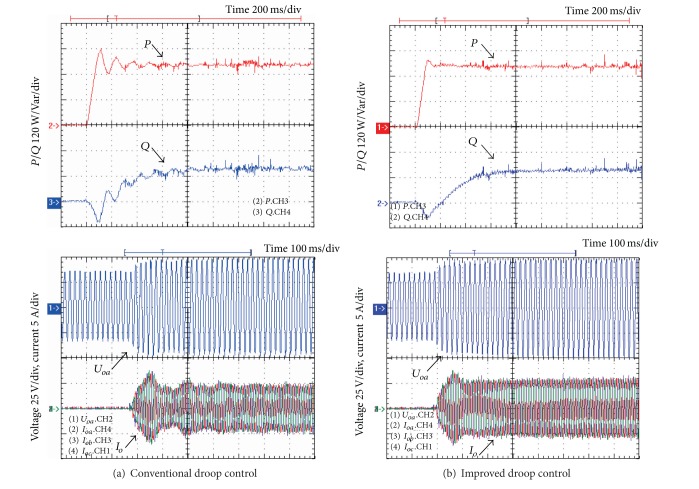
Experimental results of transient responses.

In order to verify the theoretical analysis of the small signal model, the continuous-domain simulations in MATLAB and digital control experiments based on a 32-bit fixed-point TMS320F2812 DSP are carried out. The system parameters are given as follows. Active and reactive power setpoints are 300 W and 150 W. *k*_*pp*_ = 0.1667, *k*_*qp*_ = 0.0136, *k*_*qi*_ = 0.33, *m*_*d*_ = 0.0053, *m*_*q*_ = −0.0001. Figures 7 and 8 show the simulation and experimental results of system dynamic responses, respectively.

**Figure 9:**
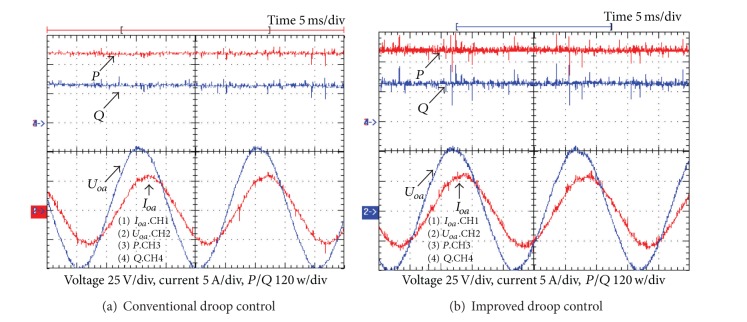
Experimental results of steady-state performance.

From the simulation and experimental results, it is clear that the poor damping and slow transient response of the conventional droop control can be mitigated with the improved solution. On the other hand, they have similar steady-state performance, as shown in Figure 9. That is to say, the improved power flow control can mitigate the problems of the conventional one without affecting the steady-state power flow regulation.

## 4. Conclusion

This paper has presented the small-signal model of the conventional and new droop control for grid-connected inverter in microgrid applications. Based on the small-signal model and the system eigenvalues, it can be concluded that the problems of the poor damping and slow transient response in conventional droop control can be solved by the improved one without affecting the steady-state power flow regulation. Finally, the simulation and experimental results verify the small signal model analysis and effectiveness of the proposed method.

## Figures and Tables

**Table 1:** Brief comparison of eigenvalues.